# Autophagy-related gene 7 is downstream of heat shock protein 27 in the regulation of eye morphology, polyglutamine toxicity, and lifespan in *Drosophila*

**DOI:** 10.1186/1423-0127-19-52

**Published:** 2012-05-23

**Authors:** Shih-Fen Chen, Ming-Lun Kang, Yi-Chun Chen, Hong-Wen Tang, Cheng-Wen Huang, Wan-Hua Li, Chun-Pu Lin, Chao-Yung Wang, Pei-Yu Wang, Guang-Chao Chen, Horng-Dar Wang

**Affiliations:** 1 Institute of Biotechnology, National Tsing Hua University, 101, Section 2, Kuang-Fu Road, HsinChu, 30013, Taiwan; 2 Department of Life Science, National Tsing Hua University, 101, Section 2, Kuang-Fu Road, HsinChu, 30013, Taiwan; 3 Institute of Biological Chemistry, 128, Section 2, Academia Road, Nankang, Taipei, 115, Taiwan; 4 Institute of Neuroscience, National Chengchi University, 64, Section 2, Zhi-Nan Road, Taipei, 11605, Taiwan; 5 Second Section of Cardiology, Department of Internal Medicine, Chang Gung Memorial Hospital at Linkou, Chang Gung University College of Medicine, Taoyuan, Taiwan

**Keywords:** Atg7, Hsp27, Neurodegeneration, Lifespan, *Drosophila*

## Abstract

**Background:**

Autophagy and molecular chaperones both regulate protein homeostasis and maintain important physiological functions. *Atg7* (*autophagy-related gene 7*) and *Hsp27* (*heat shock protein 27*) are involved in the regulation of neurodegeneration and aging. However, the genetic connection between *Atg7* and *Hsp27* is not known.

**Methods:**

The appearances of the fly eyes from the different genetic interactions with or without polyglutamine toxicity were examined by light microscopy and scanning electronic microscopy. Immunofluorescence was used to check the effect of *Atg7* and *Hsp27* knockdown on the formation of autophagosomes. The lifespan of altered expression of *Hsp27 *or *Atg7* and that of the combination of the two different gene expression were measured.

**Results:**

We used the *Drosophila* eye as a model system to examine the epistatic relationship between *Hsp27* and *Atg7*. We found that both genes are involved in normal eye development, and that overexpression of *Atg7* could eliminate the need for *Hsp27* but *Hsp27* could not rescue *Atg7* deficient phenotypes. Using a polyglutamine toxicity assay (41Q) to model neurodegeneration, we showed that both *Atg7* and *Hsp27* can suppress weak, toxic effect by 41Q, and that overexpression of *Atg7* improves the worsened mosaic eyes by the knockdown of *Hsp27* under 41Q. We also showed that overexpression of *Atg7* extends lifespan and the knockdown of *Atg7* or *Hsp27* by RNAi reduces lifespan. RNAi-knockdown of *Atg7* expression can block the extended lifespan phenotype by *Hsp27* overexpression, and overexpression of *Atg7* can extend lifespan even under *Hsp27* knockdown by RNAi.

**Conclusions:**

We propose that *Atg7* acts downstream of *Hsp27* in the regulation of eye morphology, polyglutamine toxicity, and lifespan in *Drosophila*.

## Background

The aging process results from imbalanced homeostasis combined with accumulating macromolecular damage due to different intrinsic and environmental stresses [1-3]. Protein homeostasis is important in maintaining physiological function to protect against cellular degeneration [4]. Autophagy and molecular chaperones are two defensive systems utilized to uphold cellular protein quality and homeostasis [5,6].

Macroautophagy (herein called autophagy) is a cellular, catabolic process that breaks down and recycles macromolecules and organelles under starvation conditions. Autophagy function is executed by a series of autophagy related genes (*Atg*) which are evolutionarily conserved from yeast to mammals [7]. Autophagy participates in many physiological functions including aging and neurodegeneration [8,9], and mounting evidence demonstrates that autophagy participates in the regulation of lifespan in different species [10-12]. In *C. elegans*, loss-of-function of *bec-1*/*Atg6* or RNA interference-mediated depletion of *Atg-7* or *Atg-12* inhibits the extended lifespan in *daf-2* mutants [13,14], and the knockdown of *bec-1* or *Atg7* by RNAi abolishes dietary restriction-mediated longevity in *eat-2* mutants [15]. In addition, mutations in *Atg1Atg7Atg18*, and *bec-1* reduce lifespan in *C. elegans*[16]. In *Drosophila, Atg7*-null mutants are short-lived and hypersensitive to starvation and oxidative stress [17], and the neuronal overexpression of *Atg8a* regulates lifespan and tolerance to oxidative stress [18]. *Atg7* is an E1-like enzyme and is important for the membrane elongation of the autophagosome [7]. *Atg7* deficient mice exhibit polyubiquitinated protein accumulation and neurodegeneration [19] and higher levels of polyubiquitinated proteins have been detected in the aging *Atg7* mutant fly head [17]. Autophagy also protects against neurodegeneration [20] and the induction of autophagy by the reduction of TOR (target of rapamycin) activity reduces polyglutamine toxicity in both fly and mouse [21]. Suppression of basal autophagy in the central nervous system causes neurodegeneration in *Atg7* conditional knockout mice [19,22].

Molecular chaperones modulate protein re-folding and facilitate the degradation of denatured proteins. Molecular chaperones are also implicated in several physiological functions: autophagy, neurodegeneration, stress tolerance, and aging [23-25]. Heat shock protein 27 (Hsp27) is a member of the ATP-independent, small heat shock protein family. *Hsp27* null mutants exhibit decreased lifespan and reduced starvation tolerance [26], while the overexpression of *Hsp27* increases lifespan and enhances stress resistance in *Drosophila*[27,28]. Overexpression of *Hsp27* prevents cellular polyglutamine toxicity and rescues the mosaic eyes induced by mild polyglutamine toxicity [27,29].

Both *Hsp27* and *Atg7* are involved in maintaining protein quality and modulating lifespan and neurodegeneration. However, the interaction between Hsp27 and Atg7 is unknown. We report here that Atg7 is downstream of Hsp27 in the regulation of eye morphology, polyglutamine toxicity, and lifespan in *Drosophila*. The levels of Hsp27 and Atg7 both regulate eye morphology and the polyglutamine toxicity of 41Q. The overexpression of *Atg7* rescues both the rough eye phenotype resulting from knockdown of *Hsp27* as well as the more severe mosaic eye phenotype induced by the knockdown of *Hsp27* under 41Q toxicity. In addition, the expression of *Atg7* regulates lifespan in *Drosophila* and the enhanced lifespan seen with the overexpression of *Hsp27* requires the expression of *Atg7*. Together we provide several lines of genetic evidence linking Hsp27 to Atg7 in the modulation of eye morphology, polyglutamine toxicity, and lifespan regulation.

## Methods

### Fly strains and maintenance

The RNAi lines were obtained from Vienna *Drosophila* RNAi Center (VDRC), *UAS-hsp27*^*RNAi*^ (#40530), *UAS-hsp22*^*RNAi*^ (#43632), *UAS-atg1*^*RNAi*^ (#16133), *UAS-atg4*^*RNAi*^ (#107317), *UAS-atg5*^*RNAi*^ (#104461), *UAS-atg7*^*RNAi*^ (#45560), *UAS-atg8a*^*RNAi*^ (#43096), *UAS-atg8a*^*RNAi*^ (#43097), *UAS-atg9*^*RNAi*^ (#10045), *UAS-atg12*^*RNAi*^ (#102362), *UAS-atg18*^*RNAi*^ (#105366). *GMR-Gal4; UAS-41Q* and *GMR-Gal4/Cyo; UAS-63Q* were provided by Dr. Parsa Kazemi-Esfarjani. To generate *UAS-Atg7* transgenic flies, the EST clone RE27292 containing the full-length *Atg7* was used to amplify the coding sequence by the primers (forward: 5’-GTA**CTCGAG**AAGCAA AACATGAGCACGG-3’ and reverse: 5’-CAT**AGATCT**ATCCTCGTCGCT ATCGGA-3’) and subcloned into the *XhoI* and *BglII* sites of the transgenic vector, *pINDY6*[28]. The resultant construct was verified by DNA sequencing to confirm that no mutations derived from PCR amplification were made, and injected into *w*^*1118*^ eggs for the generation of *UAS-Atg7* transgenic flies. All flies were maintained on standard fly food as described in Liu *et al.*[30] and incubated at 25°C, 65% humidity, in a 12 h/12 h light/dark-cycle fly incubator.

### Fly eye image

Two-day-old flies of the different types were anaesthetized by carbon dioxide on a porous platform and the eye images were taken by light microscopy (SMZ1500, Nikon). For the scanning electron micrograph, the fly was fixed on a copper stage and the fly eye image was acquired by scanning electron microscopy (TM-1000, Hitachi). For each fly line, a total of more than 86 eye images from at least three independent crosses were examined.

### RT-PCR and real-time PCR

Total RNA was prepared from about 20 flies of each specific allele and homogenized in 1 ml Trizol solution. Equal amounts (1 μg) of each DNase I-treated RNA were reverse-transcribed to cDNA with MMLV reverse transcriptase (Promega). The cDNAs were used as templates for RT-PCR or real-time PCR as described in Liu *et al.*[30]. The information of the primers is available upon request.

### Lifespan and starvation assays

For the lifespan assay, all the fly lines have been outcrossed with *w*^*1118*^ as described previously [31]. The newly eclosed flies of each allele were collected by sex with 30 flies per vial, maintained at 25°C, 65% humidity in a 12 h/12 h light/dark-cycle fly incubator and transferred to a new vial every 3 or 4 days until all were dead. The statistical significance was calculated by log rank test. At least three independent measurements were performed for each experiment.

For the starvation assay, newly eclosed flies of each type were collected by sex with 20 flies per vial and recovered overnight. Next day the flies were transferred to the vials with 1% agar and transferred to new agar vials daily. The numbers of the dead flies were recorded every 4 hours until all were dead. The statistical significance was calculated by student’s *t* test.

### Immunofluorescence

GFP-NLS-marked Atg7 or Hsp27 RNAi knockdown clones in the larval fat body were generated by heat shock-independent FLP/FRT induction as described previously [32,33]. FLP/FRT method allows to examine the mitotic GFP-NLS-marked RNAi knockdown clones surrounded by the control cells that do not incorporate the RNAi knockdown in the same tissue under the same condition [33]. Fat bodies from early third instar larva cultured in standard fly food with yeast paste (fed condition) or in dishes containing 20% sucrose only (starvation condition) for 4 hr were dissected and fixed with 4% paraformaldehyde and then examined by confocal laser scanning microscope (LSM510; Carl Zeiss Inc.) equipped with a 63x Plan-Apochromat (NA1.4) objective lens.

## Results

### *Autophagy-related gene 7* is downstream of *heat shock protein 27* in the regulation of *Drosophila* eye phenotype

**Figure 1:**
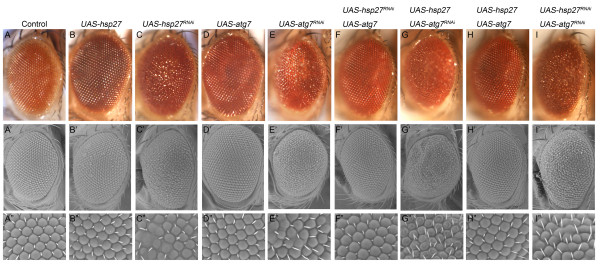
**Atg7 Is Downstream of Hsp27 in the Regulation of*Drosophila*Eye Phenotype.** (**A**-**A”**) The *GMR-Gal4/+* control fly has a normal eye phenotype and normal shape of individual ommatidia. (**B**-**B’**, **D**-**D”**) Overexpression of *Hsp27* or *Atg7* also results in a normal eye phenotype and regular ommatidia. (**C**-**C”**, **E**-**E”**) Knockdown of *Hsp27* or *Atg7* displays similar rough eye phenotype and enlarged and fused ommatidia. (**F**-**F”**) Overexpression of *Atg7* rescues the rough eye and irregular shape of ommatidia by knockdown of *Hsp27*. (**G**-**G”**) Overexpression of *Hsp27* cannot revert the rough eye and abnormal shape of ommatidia resulting from the knockdown of *Atg7*. (**H**-**H”**) Co-overexpression of *Hsp27* and *Atg7* still leads to normal eye phenotype and ommaditia. (**I**-**I”**) Co-knockdown of *Hsp27* and *Atg7* causes a similar phenotype: rough eyes and irregular shape of ommatidia like that of the individual knockdowns of *Hsp27* or *Atg7*. Optical micrograph (A-I) and scanning electron micrograph (A’-I’: 300X; A”-I”:1500X). Genotypes: *GMR-Gal4* in *trans* to the alleles indicated.

Protein homeostasis plays an important role in lifespan and stress response [1,2]. Heat shock protein 27 (Hsp27) has been shown to regulate lifespan and response to different stresses [26-28]. *Autophagy-related gene 7* (*Atg7*) is required for normal lifespan and tolerance to starvation and oxidation [17]. However, the genetic interaction between *Hsp27* and *Atg7* is unknown. We examined the effects of altering *Hsp27* and *Atg7* expression in the *Drosophila* eye using the *GMR-Gal4* driver followed by the analyses of eye morphology utilizing scanning electron microscopy and light microscopy. Overexpression of *Hsp27* or *Atg7* results in a normal eye phenotype and regular ommatidia shape as seen in the *GMR-Gal4* control flies (Figure 1, A-A”, B-B”, D-D”). Interestingly, knockdown expression of either *Hsp27* or *Atg7* by expression of interfering RNAs using *GMR-Gal4* results in similar rough eye phenotypes with fused and enlarged ommatidia (Figure 1, C-C”, E-E”). Overexpression of *Atg7* in the *Hsp27* knockdown background fully rescues the rough eye phenotype of the *Hsp27* knockdown (Figure 1, F-F”). However, overexpression of *Hsp27* in the *Atg7* knockdown background fails to rescue the rough eye phenotype of the *Atg7* knockdown (Figure 1, G-G”). These results suggest that *Atg7* is located downstream of *Hsp27* in the regulation of *Drosophila* eye morphology. To further confirm that *Hsp27* and *Atg7* function in the same pathway controlling eye phenotype, we examined whether there is any additive effect on fly eye morphology by either the co-overexpression or co-knockdown of *Hsp27* and *Atg7*. The overexpression of both *Hsp27* and *Atg7* in combination produces a normal eye phenotype, similar to the overexpression of *Hsp27* or *Atg7* alone (Figure 1, B-B”, D-D”, H-H”). The simultaneous knockdown of *Hsp27* and *Atg7* does not further deteriorate the rough eye phenotype when compared to the effects of either gene alone (Figure 1, C-C”, E-E”, I-I”), implying that *Hsp27* and *Atg7* operate in the same pathway. These data provide the first evidence that *Atg7* is downstream of *Hsp27* in the regulation of *Drosophila* eye morphology.

### Knockdown of other *autophagy-related genes* and *heat shock protein 22* does not result in a rough eye phenotype in *Drosophila*

**Figure 2:**
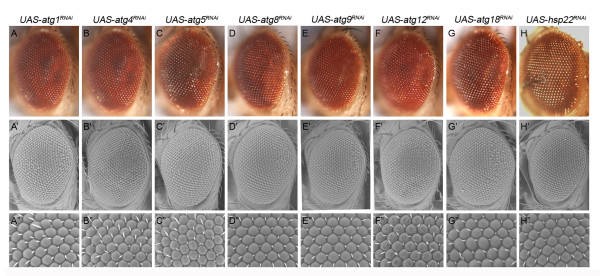
**Knockdown of the Other Autophagy-related Genes and Heat Shock Protein 22 Do Not Result in Any Rough Eye Phenotype in*Drosophila*.** RNAi knockdown of different autophagy-related genes by *GMR-Gal4* show normal eye morphology and regular ommatidia (like the control in Figure 1, A-A”. (**A**-**A”**) Atg1, (**B**-**B”**) Atg4, (**C**-**C”**) Atg5, (**D**-**D”**) Atg8a, (**E**-**E”**) Atg9, (**F**-**F”**) Atg12, (**G**-**G”**) Atg18, and (**H**-**H”**) Hsp22. Optical micrograph (A-H) and SEM (A’-H’:300X; A”-H”:1500X). Genotypes: *GMR-Gal4* in *trans* to the alleles indicated.

To determine whether the rough eye phenotype is specific to *Atg7*, or whether it represents a general effect of altering autophagy, the effects of knockdown of additional autophagy-related genes were examined by using *GMR-Gal4* and none of these displayed the rough eye phenotype (Figure 2, Figure 1, E-E”). Knockdown of *Atg1* shows a normal eye phenotype (Figure 2, A-A”, Figure 1, A-A”), while knockdown of other autophagy-related genes: *Atg 4, 5, 8, 9, 12, 18* displayed subtle eye color phenotypes but had no effect on the ommatidia structure (Figure 2, B-G, B’-G’, B”-G”). These data suggests that the rough eye phenotype resulting from *Atg7* knockdown is *Atg7*-specific and not involved in the alteration of other autophagy-related genes. Similarly, to examine whether the rough eye phenotype is specific to *Hsp27* knockdown, we tested the effects of knockdown of *Hsp22*, another known lifespan modulation gene [34], by *GMR-Gal4* and did not observe any effects on the eye like that of *Hsp27* knockdown (Figure 1, C-C”). Q-PCR analysis confirms that there is reduced expression of *Atg* and *Hsp22* genes in the RNAi knockdown experiments (data not shown). Thus the rough eye phenotype is specific to the knockdown of either *Atg7* or *Hsp27*.

### Knockdown of *Atg7* but not *Hsp27* blocks starvation-induced autophagosome formation

**Figure 3:**
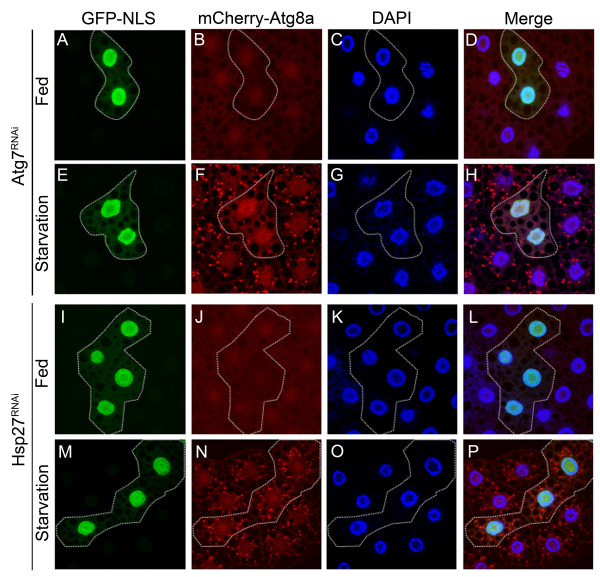
**Starvation-induced Autophagosome Formation is Inhibited by RNAi-mediated Depletion of Atg7 but not Hsp27.** (**A**, **E**, **I**, **M**) GFP-NLS labeled fat body cells circled by dotted line indicate the presence of *UAS-Atg7*^*RNAi*^or *UAS-Hsp27*^*RNAi*^generated by the FLP/FRT method. The cells outside of the circled dotted line are used as the control cells without *UAS-Atg7*^*RNAi*^or *UAS-Hsp27*^*RNAi*^. (**B**, **F**, **J**, **N**) The distribution patterns of mcherry-Atg8a are shown under either fully-fed or starvation conditions. (**C**, **G**, **K**, **O**) The fat body cells are stained with DAPI. (**D**, **L**) The picture D is merged from panels A, B, C and L is merged from panels I, J, K under nutrient-rich conditions. (**H**, **P**) Picture H is merged from panels E, F, G and P is merged from M, N, O under starvation conditions. The distribution of mcherry-Atg8a puncta is dramatically altered in starved fat body cells (F, N) compared to those under nutrient-rich conditions (B, J). GFP-labeled cells expressing *Atg7-RNAi* markedly suppress mCherry-Atg8a puncta formation (F), but not in that of *Hsp27* knockdown (N).

To verify that the knockdown of *Atg7* by *UAS-Atg7*^*RNAi*^ from VDRC can affect starvation-induced autophagy, we generated *UAS-Atg7*^*RNAi*^ clones in the fat-body by using the FLP/FRT method [32,33] and examined the distribution of mcherry-Atg8a puncta. The distribution of mcherry-Atg8a is in a uniformly diffuse structure under optimal feeding conditions (Figure 3, B, J) and becomes localized to punctate structure under starvation conditions (Figure 3, F, N). Under starvation conditions, the GFP-NLS clones with the *Atg7* knockdown, where the cells are circled by dotted line, display a reduced number of mcherry-Atg8a puncta than the surrounding control clones without *Atg7* knockdown which have no GFP-NLS signal (Figure 3, E, F). These results demonstrate that knockdown of *Atg7* by *UAS-Atg7*^*RNAi*^ is able to block mcherry-Atg8a mediated autophagosome formation under starvation. To examine whether knockdown of *Hsp27* can alter autophagosome formation, we also generated *UAS-Hsp27*^*RNAi*^ clones in the fat body and inspected the distribution of mcherry-Atg8a puncta. Under starvation, the autophagosome formation indicated by mcherry-Atg8a puncta is not altered by comparing the GFP-NLS marked *Hsp27* RNAi knockdown clones, which are circled by dotted line, to the surrounding control clones without GFP-NLS signal and no *Hsp27* RNAi knockdown (Figure 3, M, N). The data indicate that *Hsp27* knockdown does not block the mcherry-Atg8a mediated autophagosome formation under starvation. The notion is consistent with the previous data since knockdown of *Atg8* does not result in the rough eye as the knockdown of *Hsp27*, suggesting that *Hsp27* and *Atg8* do not function in the same genetic pathway.

### *Atg7* and *Hsp27* attenuate the mild polyglutamine toxicity of 41Q but cannot rescue longer polyglutamine tract toxicity by 63Q

**Figure 4:**
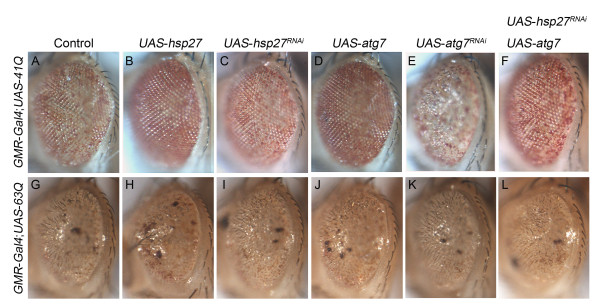
***Atg7*is Downstream of*Hsp27*in the Attenuation of the Mild Polyglutamine Toxicity by 41Q, but the Overexpression of Both Genes Cannot Rescue the Longer Polyglutamine Tract Toxicity by 63Q.** (**A**) Expression of *UAS-41Q* by *GMR-GAL4* results in mosaic eyes. (**B**, **D**) Both the overexpression of *Hsp27* and *Atg7* rescue the mosaic eye by 41Q. (**C**, **E**) Under 41Q background, both the knockdown of *Hsp27* and *Atg7* generate comparable worsened mosaic eyes, whereas knockdown of *Atg7* leads to a rough eye surface. (**F**) Overexpression of *Atg7* as well as knockdown of *Hsp27* improves the mosaic eye by 41Q. (**G**, **M**) The expressions of *UAS-63Q* by *GMR-Gal4* produce similar rough eye phenotype. (**H**, **J**) Overexpression of *Hsp27* or *Atg7* cannot rescue the rough eye induced by 63Q. (**I**, **K**) Knockdown of *Hsp27* or *Atg7* in conjunction with 63Q does not cause further deterioration of the eyes. (**L**) Overexpression of *Atg7* together with knockdown of *Hsp27* does not alter the rough eye phenotype by 63Q. Genotypes: (A-F) *GMR-Gal4; UAS-41Q* in *trans* to the alleles indicated. (G-L) *GMR-Gal4/Cyo; UAS-63Q* in *trans* to the alleles indicated.

Overexpression of *Hsp27* can rescue the mosaic eye phenotype resulting from mild polyglutamine (41Q)-induced toxicity but not the rough eye phenotype resulting from severe polyglutamine (127Q) toxicity [27]. Since *Atg7* acts downstream of *Hsp27* in the eye, we were interested in determining whether the overexpression of *Atg7* would also only rescue mild polygutamine phenotypes. As with *Hsp27*, the overexpression of *Atg7* rescues the mosaic eye phenotype caused by 41Q (Figure 4, A, B, D) but cannot rescue the more severe, rough eye phenotypes resulting from the longer polyglutamine tract of 63Q (Figure 4, G, H, J). The knockdown of either *Hsp27* or *Atg7* enhances the pigmentation phenotype observed in the eye of flies expressing 41Q. Interestingly only the knockdown of *Atg7,* but not that of *Hsp27,* enhances the eye morphology phenotype (rough eye) in combination with 41Q overexpression (Figure 4, C, E). The knockdown of *Hsp27* or *Atg7* does not exacerbate the rough eye phenotypes of 63Q (Figure 4, I). Interestingly, the overexpression of *Atg7* partially rescues the more dramatic mosaic eye phenotype induced by *Hsp27* knockdown in the 41Q background (Figure 4, C, F), supporting the idea that *Atg7* is downstream of *Hsp27* in the alleviation of 41Q toxicity. However, the combination of the overexpression of *Atg7* and knockdown of *Hsp27* do not change the rough eye phenotype of 63Q (Figure 4, L).

### *Atg7* regulates lifespan and is required for *Hsp27*-mediated extended lifespan in *Drosophila*

**Figure 5:**
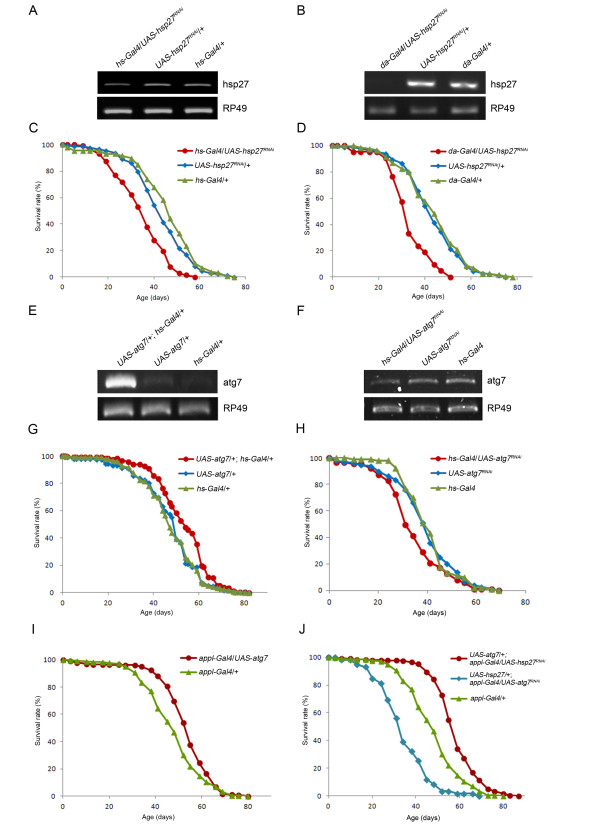
**Atg7 Is Downstream of Hsp27 in the Regulation of*Drosophila*Lifespan.** (**A**, **B**, **E**, **F**) RT-PCR verifies that the transcript levels of *Hsp27* and *Atg7* are altered upon Gal4 induction. (**C**, **D**) RNAi knockdown of *Hsp27* by *hs-Gal4* and *da-Gal4* both reduce *Drosophila* lifespan. (**G**) Overexpression of *Atg7* by *hs-Gal4* increases *Drosophila* lifespan. (**H**) Knockdown of *Atg7* by *hs-Gal4* decreases *Drosophila* lifespan. (**I**) Neuronal overexpression of *Atg7* by *appl-Gal4* enhances *Drosophila* lifespan. (**J**) Overexpression of *Atg7* along with knockdown of *Hsp27* by *appl-Gal4* displays extended lifespan. On the other hand, simultaneous knockdown of *Atg7* and overexpression of *Hsp27* exhibits reduced lifespan.

Hsp27 levels are likely to regulate *Drosophila* lifespan since *Hsp27* overexpression extends *Drosophila* lifespan [27,28] while the knockout *Hsp27* mutant is short-lived [26]. The knockdown of *Hsp27* by either *hs-Gal4,* or *da-Gal4* exhibits reduced *Hsp27* levels and displays a 20% (*P* < 0.001), and 27% (*P* < 0.001) decrease in mean lifespan, respectively (Figure 5, A - D; Additional file 1: Table S1). Since *Atg7* is downstream of *Hsp27* in the regulation of eye morphology and mild polyglutamine toxicity, and *Atg7* null mutants display shortened lifespan [17], we tested whether *Hsp27*-mediated enhanced lifespan requires *Atg7*. *Atg7* overexpression by *hs-Gal4* shows a robust increase in *Atg7* transcripts relative to control flies and increases the mean lifespan by about 11% (*P* < 0.01) relative to the control flies (Figure 5, E and G; Additional file 2: Table S2). Conversely, knockdown of *Atg7* by *hs-Gal4* exhibits reduced levels of *Atg7* transcripts and decreases mean lifespan by about 10% (*P* < 0.01) when compared to the control flies (Figures 5F and H; Additional file 2: Table S2). These results indicate that like *Hsp27Atg7* levels also regulate *Drosophila* lifespan.

It has been shown that neuronal overexpression of *Atg8a* by *appl-Gal4* extends *Drosophila* lifespan and increases resistance to starvation [18]. To test whether neuronal overexpression of *Atg7* enhances lifespan and starvation resistance, *Atg7* was overexpressed in neurons using *appl-Gal4*, resulting in increases of 12% (*P* < 0.001) in mean lifespan and 18% (*P* < 0.01) in starvation resistance (Figure 5, I; Additional file 2: Table S2 and Additional file 3:Table S4). In addition, the simultaneous overexpression of *Atg7* and knockdown of *Hsp27* results in flies that exhibit a 21% (*P* < 0.001) extension in mean lifespan (Figure 5, J). Conversely, the flies possessing both knockdown of *Atg7* and overexpressing *Hsp27* display a reduction of 27% (*P* < 0.001) in mean lifespan relative to the control flies (Figure 5, J; Additional file 4 : Table S3). To further demonstrate that *Atg7* functions downstream of *Hsp27*, we carried out the locomotion assay to measure the climbing activity of the flies with the different combination of overexpression and knockdown of *Atg7* and *Hsp27* along with the control flies under paraquat-induced oxidative stress. Similar to the lifespan result, the flies with simultaneous overexpression of *Atg7* and knockdown of *Hsp27* displayed significantly better climbing activity (42%, *P* ≤ 0.001) than that of the control flies (22%), and the flies with simultaneous knockdown of *Atg7* and overexpression of *Hsp27* exhibited a significantly lowered locomotion activity (15%, *P* ≤ 0.01) than that of the control flies (Additional file 5 : Figure S1). The climbing activity data in accordance with the lifespan data supports our hypothesis that *Atg7* acts downstream of *Hsp27*. Taken together, these results indicate that as seen with *Drosophila* eye morphology and polyglutamine toxicity, *Atg7* also acts downstream of *Hsp27* in regulating lifespan.

## Discussion

*Hsp27* and *Atg7* are both implicated in the processes of aging and neurodegeneration. In this report, we provide several lines of evidence to show that *Atg7* is downstream of *Hsp27* in the regulation of eye morphology, polyglutamine toxicity, and lifespan. Autophagy-related genes are conserved among different species [7,35]. Each of the identified *Atgs* has a role in autophagy, but their roles in other processes remains largely unclear.

In the examination of eye phenotype, we observed that the knockdown of either *Hsp27* or *Atg7* exhibited similar rough eye phenotypes. These effects appear to be specific to these particular molecules since the knockdown of other *Atgs* (*Atg1Atg4Atg5Atg8aAtg9Atg12and Atg18*) or *Hsp22* does not produce a similar, rough eye phenotype. The ability of *Atg7* to rescue the phenotype induced by *Hsp27* knockdown also suggests that a unique interaction exists between Hsp27 and Atg7. A recent study indicates that knockdown of *Atg7* by *GMR-Gal4* on X chromosome causes retinal degeneration [36]. In addition, the rhabdomeres were shown degenerated in the aged *atg7*^*d77*^ mutant flies [37]. Both support our finding that RNAi knockdown of *Atg7* results in rough eye in *Drosophila*.

Autophagy serves to protect against neurodegenerative diseases [20] and aberrations in autophagy have been implicated in neurodegeneration [38]. In both fly and mouse models, induction of autophagy by inhibiting mTOR ameliorates polyglutamine toxicity [21]. And in humans, a polymorphism study of more than 900 European Huntington’s disease patients revealed that one variant of *Atg7* (*Atg7*^*V471A*^) is statistically correlated with early onset of Huntington’s disease [39]. These findings suggest that a specific function of *Atg7* is to attenuate polyglutamine toxicity and support our findings that *Atg7* rescues polyglutamine toxicity by 41Q in *Drosophila*. Hsp27 has also been shown to reduce cellular polyglutamine toxicity [29] and the overexpression of *Hsp27* in *Drosophila* rescues the pigmentation defects induced by 41Q [27]. Several lines of evidence suggest that heat shock proteins may rely upon autophagy to reduce polyglutamine toxicity. For example, the anti-polyglutamine-aggregation activity of HspB7, one of the human small heat shock proteins, was substantially diminished in *Atg5*-deficient cells [40]. In addition, it is possible that the small heat shock protein HspB8-Bag3 complex enhance Htt43Q degradation via autophagy since the treatment of the Htt43Q transfected HEK-293T and COS1 cells with an autophagy inhibitor significantly reduced HspB8-Bag3-mediated Htt43Q degradation [41]. Furthermore, it was recently suggested that the small heat shock protein HspB7 assists in the loading of misfolded proteins or aggregates in autophagosomes [42]. Together, these findings indicate that autophagy is downstream of small heat shock proteins and support our results that *Atg7* is downstream of *Hsp27*.

The inhibition of autophagy results in decreased lifespan. Atg7 activity is essential for the longevity resulting from either reduced insulin signaling or caloric restriction in which depletion of *Atg7* was found to block the longevity phenotypes of both *daf-2* and *eat-2* mutants [13,15]. Our data showed that RNAi knockdown of *Atg7* by *hs-Gal4,* starting from embryonic to adulthood stage, results in a shortened lifespan similar to that of the *Drosophila Atg7* null mutant [17]. Loss-of-function mutations in *Atg7* as well as *Atg1Atg18*, and *Beclin-1* shorten lifespan in *C. elegans*[16]. Several autophagy mutants including *Atg7* were identified chronologically short-lived in a yeast genetic screen [43]. However, it should be noted that not all autophagy genes are linked to aging and *Atg7* is one of the conserved *Atg* genes that is involved in the regulation of aging in most species [9]. Conversely, the induction of autophagy increases lifespan. The induction of autophagy by caloric restriction or reducing target of rapamycin (TOR) activity enhances lifespan [9] and the neuronal overexpression of *Atg8a* increases *Drosophila* lifespan [18]. We have found that the overexpression of *Atg7* extends lifespan in *Drosophila* and that the neuronal overexpression of *Atg7* is sufficient to reverse *Hsp27*-knockdown-mediated, shortened lifespan. Knockdown of *Atg7* blocks Hsp27-mediated extended lifespan, again supporting the model that Atg7 acts downstream of Hsp27 in the regulation of lifespan. It has been reported that in adult flies, RNAi knockdown of *Atg7* by *Geneswitch-Actin-Gal4* did not show reduced lifespan [44]. This discrepancy may be due to the different Gal4 drivers used and that the knockdown of *Atg7* occurring only during adulthood is insufficient to cause shortened lifespan since autophagy activity is known to be tightly regulated during development.

Yet we cannot exclude that chaperone-mediated autophagy (CMA) is involved in the connection between Hsp27 and Atg7. CMA is a specific cargo delivery process to the lumen of the lysosome, mediated by Hsc70, Hsp90, and the lysosome-associated membrane protein type 2A (LAMP-2A) [45,46]. However, a recent study in *Drosophila* shows that the co-chaperone Starvin assists in the coordination of Hsc70 and HspB8 through chaperone-assisted selective autophagy, which is distinct from CMA, to depose damaged filamin for muscle maintenance [47]. It is possible that Hsp27 may function through chaperone-assisted selective autophagy linking to Atg7.

## Conclusion

In summary, our finding sheds new insight in the linkage of Hsp27 to Atg7 in the regulation of eye morphology, polyglutamine toxicity, and lifespan. The information provides a new aspect in the understanding how Hsp27 may connect to Atg7 to modulate certain physiological functions.

## Abbreviations

Atg, autophagy-related gene; Hsp, heat shock protein.

## Competing Interests

The authors declare that they have no competing interests.

## Authors’ contributions

S-F Chen, M-L Kang, Y-C Chen, H-W Tang, C-W Huang, W-H Li, and C-P Lin carried out the experiments and analyzed the data; P-Y Wang, G-C Chen and H-D Wang designed the experiments, analyzed the data, and together with C-Y Wang discussed the data and wrote the manuscript. All authors read and approved the final manuscript.

## Supplementary Material

Additional file 1 **Table S1.** A summary of lifespan by the knockdown of *Hsp27* in *Drosophila.* Click here for file

Additional file 2 **Table S2.** A summary of lifespan by the overexpression or knockdown of *Atg7* in *Drosophila.* Click here for file

Additional file 3 **Table S4.** A summary of starvation stress response by overexpression of *Atg7* in *Drosophila.* Click here for file

Additional file 4 **Table S3.** A summary of lifespan resulting from simultaneous overexpression and knockdown of different combinations of *Atg7* and *Hsp27* in *Drosophila.* Click here for file

Additional file 5 **Figure S1.** The flies with simultaneous overexpression of *Atg7* and knockdown of *Hsp27* display better climbing activity than those with overexpression of *Hsp27* and knockdown of *Atg7* under paraquat-induced oxidative stress. The climbing index for each strain: *appl-Gal4/+*(the control fly): 21.8 ± 0.02% (n = 195); *UAS-hsp27/+; appl-Gal4/UAS-atg7*^*RNAi*^: 14.7 ± 0.02% (n = 123); *UAS-atg7/+; appl-Gal4/UAS-hsp27*^*RNAi*^: 42.4 ± 0.01% (n = 175). (n is the total fly number from the four independent assays.). (**p < 0.01, ***p < 0.001). Click here for file
