# Supplementary material for: Autophagy-related gene 7 is downstream of heat shock protein 27 in the regulation of eye morphology, polyglutamine toxicity, and lifespan in Drosophila
Source: J Biomed Sci. 2012 May 23;19(1):52. doi: 10.1186/1423-0127-19-52 (PMC3483682; doi:10.1186/1423-0127-19-52)
Supplement: Additional file 1 — Table S1. A summary of lifespan by the knockdown of Hsp27 in Drosophila. [file 1423-0127-19-52-S1.docx]

**Table S1.** A summary of lifespan by the knockdown of *Hsp27* in *Drosophila*.

| **Lifespan** | |  | | |  |  |  | |
| --- | --- | --- | --- | --- | --- | --- | --- | --- |
| Strain ♂ | Sample size | | Mean (days) | Difference,% compare to (UAS/+) | | Difference,% compare to (Gal4/+) | |  |
| *hs-Gal4/UAS-hsp27^RNAi^* | 143 | | 34.6 | -20.3*** | | -24.5*** | |  |
| *UAS-hsp27^RNA^*^i^/+ | 177 | | 43.4 |  | |  | |  |
| *hs-Gal4*/+ | 158 | | 45.8 |  | |  | |  |
| *P*-value were calculated by log-rank test: ****p* < 0.001 | | | | | | | | |

| **Lifespan** | |  | | |  |  |  | |
| --- | --- | --- | --- | --- | --- | --- | --- | --- |
| Strain ♂ | Sample size | | Mean (days) | Difference,% compare to (UAS/+) | | Difference,% compare to (Gal4/+) | |  |
| *da-Gal4/UAS-hsp27^RNAi^* | 48 | | 31.3 | -27.9*** | | -27.7*** | |  |
| *UAS*-*hsp27^RNAi^*/+ | 177 | | 43.4 |  | |  | |  |
| *da-Gal4*/+ | 167 | | 43.3 |  | |  | |  |

*P*-value were calculated by log-rank test: ****p* < 0.001
