# Supplementary material for: Autophagy-related gene 7 is downstream of heat shock protein 27 in the regulation of eye morphology, polyglutamine toxicity, and lifespan in Drosophila
Source: J Biomed Sci. 2012 May 23;19(1):52. doi: 10.1186/1423-0127-19-52 (PMC3483682; doi:10.1186/1423-0127-19-52)
Supplement: Additional file 2 — Table S2. A summary of lifespan by the overexpression or knockdown of Atg7 in Drosophila. [file 1423-0127-19-52-S2.docx]

**Table S2.** A summary of lifespan by the overexpression or knockdown of *Atg7* in *Drosophila*.

| **Lifespan** | |  | | |  |  |  | |
| --- | --- | --- | --- | --- | --- | --- | --- | --- |
| Strain ♂ | Sample size | | Mean (days) | Difference,% compare to (UAS/+) | | Difference,% compare to (Gal4/+) | |  |
| *UAS*-*atg7*/+; *hs-Gal4/*+ | 244 | | 58.3 | 11.7** | | 11.0** | |  |
| *UAS*-*atg7*/+ | 211 | | 52.2 |  | |  | |  |
| *hs*-*Gal4*/+ | 244 | | 52.5 |  | |  | |  |

*P*-value were calculated by log-rank test: ***p* < 0.01

| **Lifespan** | |  | | |  |  |  | |
| --- | --- | --- | --- | --- | --- | --- | --- | --- |
| Strain ♂ | Sample size | | Mean (days) | Difference,% compare to (UAS/+) | | Difference,% compare to (Gal4/+) | |  |
| *hs*-*Gal4*/*UAS*-*atg7^RNAi^* | 84 | | 36.0 | -9.8** | | -11.8** | |  |
| *UAS*-*atg7^RNAi^*/+ | 236 | | 40.0 |  | |  | |  |
| *hs*-*Gal4*/+ | 90 | | 40.9 |  | |  | |  |

*P*-value were calculated by log-rank test: ***p* < 0.01

| **Lifespan** | |  | | |  |
| --- | --- | --- | --- | --- | --- |
| Strain ♂ | Sample size | | Mean (days) | Difference,% compare to (Gal4/+) | |
| *appl-Gal4*/*UAS*-*atg7* | 400 | | 50.0 | 12.0*** | |
| *appl*-*Gal4*/+ | 243 | | 44.6 |  | |

*P*-value were calculated by log-rank test: ****p* < 0.001
