# Supplementary material for: Autophagy-related gene 7 is downstream of heat shock protein 27 in the regulation of eye morphology, polyglutamine toxicity, and lifespan in Drosophila
Source: J Biomed Sci. 2012 May 23;19(1):52. doi: 10.1186/1423-0127-19-52 (PMC3483682; doi:10.1186/1423-0127-19-52)
Supplement: Additional file 3 — Table S4. A summary of starvation stress response by overexpression of Atg7 in Drosophila. [file 1423-0127-19-52-S3.docx]

**Table S4.** A summary of starvation stress response by overexpression of *Atg7* in *Drosophila.*

| **Starvation stress** | |  | | |  |  |  |
| --- | --- | --- | --- | --- | --- | --- | --- |
| Strain ♂ | Sample size | | Mean (hours) | Difference,% compare to (UAS/+) | | Difference,% compare to (Gal4/+) | |
| *appl-Gal4*/*UAS*-*atg7* | 194 | | 72.2 | 18.2** | | 19.8** | |
| *UAS*-*atg7* | 196 | | 61.1 |  | |  | |
| *appl*-*Gal4*/+ | 198 | | 60.3 |  | |  | |

*P*-value were calculated by log-rank test: ****p* < 0.001
