# Supplementary material for: Autophagy-related gene 7 is downstream of heat shock protein 27 in the regulation of eye morphology, polyglutamine toxicity, and lifespan in Drosophila
Source: J Biomed Sci. 2012 May 23;19(1):52. doi: 10.1186/1423-0127-19-52 (PMC3483682; doi:10.1186/1423-0127-19-52)
Supplement: Additional file 4 — Table S3. A summary of lifespan resulting from simultaneous overexpression and knockdown of different combinations of Atg7 and Hsp27 in Drosophila. [file 1423-0127-19-52-S4.docx]

**Table S3.** A summary of lifespan resulting from simultaneous overexpression and knockdown of different combinations of *Atg7* and *Hsp27* in *Drosophila.*

| **Lifespan** | |  | | |  |
| --- | --- | --- | --- | --- | --- |
| Strain ♂ | Sample size | | Mean (days) | Difference,% compare to (Gal4/+) | |
| *UAS*-*atg7*/+; *appl*-*Gal4*/*UAS*-*hsp27^RNAi^* | 322 | | 58.2 | 20.9*** | |
| *UAS*-*hsp27*/+; *appl*-*Gal4*/*UAS*-*atg7^RNAi^* | 59 | | 35.1 | -27.0*** | |
| *appl*-*Gal4*/+ | 243 | | 48.1 |  | |

*P*-value were calculated by log-rank test: ****p* < 0.001
