# Supplementary material for: Autophagy-related gene 7 is downstream of heat shock protein 27 in the regulation of eye morphology, polyglutamine toxicity, and lifespan in Drosophila
Source: J Biomed Sci. 2012 May 23;19(1):52. doi: 10.1186/1423-0127-19-52 (PMC3483682; doi:10.1186/1423-0127-19-52)
Supplement: Additional file 5 — Figure S1. The flies with simultaneous overexpression of Atg7 and knockdown of Hsp27 display better climbing activity than those with overexpression of Hsp27 and knockdown of Atg7 under paraquat-induced oxidative stress. The climbing index for each strain: appl-Gal4/+(the control fly): 21.8 ± 0.02% (n = 195); UAS-hsp27/+; appl-Gal4/UAS-atg7RNAi: 14.7 ± 0.02% (n = 123); UAS-atg7/+; appl-Gal4/UAS-hsp27RNAi: 42.4 ± 0.01% (n = 175). (n is the total fly number from the four independent assays.). (**p < 0.01, ***p < 0.001). [file 1423-0127-19-52-S5.docx]

**Figure S1.**


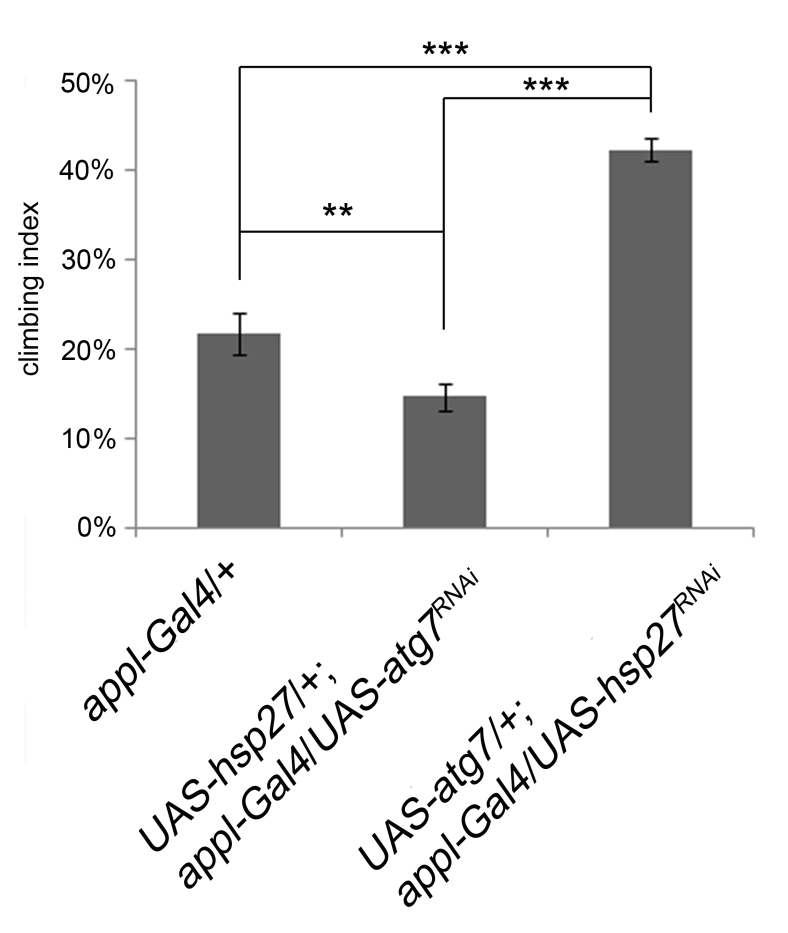


**Figure S1.** The flies with simultaneous overexpression of *Atg7* and knockdown of *Hsp27* display better climbing activity than those with overexpression of *Hsp27* and knockdown of *Atg7* under paraquat-induced oxidative stress*.* The climbing index for each strain: *appl-Gal4/+*(the control fly): 21.8±0.02% (n=195) ; *UAS-hsp27/+; appl-Gal4/UAS-atg7^RNAi^*: 14.7±0.02% (n=123) ; *UAS-atg7/+; appl-Gal4/UAS-hsp27^RNAi^*: 42.4±0.01% (n=175). (n is the total fly number from the four independent assays.). (***p* < 0.01, ****p* < 0.001).

**Supplemental Figure method**:

**Locomotion activity assay**. Three-day-old male flies of each genotype as indicated were collected and treated with 10mM paraquat in 5% sucrose solution for 24 hours. The survived flies of the different genotypes were placed to the plastic test tubes (Falcon, cat#2057) for the countercurrent apparatus for three-minute rest before the locomotion activity assay. The flies in the countercurrent apparatus were shaken down to the bottom of the test tubes and given 10 seconds to climb up to the upper tube and then slide the apparatus to collect the flies that climb over the 9.5-cm test tube. The climbing index is defined as the number of the flies climbing over the test tube divided by the total number of the flies. Four independent locomotion activity assays were carried out to measure the climbing activity. Student’s *t* test was used to calculate the statistical significance.
